# The value of single-molecule real-time technology in the diagnosis of rare thalassemia variants and analysis of phenotype–genotype correlation

**DOI:** 10.1038/s10038-021-00983-1

**Published:** 2021-10-25

**Authors:** Shiqiang Luo, Xingyuan Chen, Dingyuan Zeng, Ning Tang, Dejian Yuan, Qingyan Zhong, Aiping Mao, Ruofan Xu, Tizhen Yan

**Affiliations:** 1 Department of Medical Genetics, Liuzhou Key Laboratory of Birth Defects Prevention and Control, Liuzhou Maternity and Child Healthcare Hospital, 545001 Liuzhou, Guangxi China; 2 Liuzhou Key Laboratory of Reproductive Medicine, 545001 Liuzhou, Guangxi China; 3 Department of Laboratory Medicine, The People’s Hospital of Guangxi Zhuang Autonomous Region, Nanning, Guangxi Zhuang Autonomous Region China; 4 Guangxi Academy of Medical Sciences, Nanning, Guangxi Zhuang Autonomous Region China; 5 Guangxi Health Commission Key Laboratory of Birth Cohort Study in Pregnant Women of Advanced Age, 545001 Liuzhou, Guangxi China; 6 Berry Genomics Corporation, 102200 Beijing, China

**Keywords:** Single molecule real-time technology (SMRT), α-thalassemia rare variant, β-thalassemia rare variant, Carrier screening, Sequencing, Anaemia

## Abstract

To compare single-molecule real-time technology (SMRT) and conventional genetic diagnostic technology of rare types of thalassemia mutations, and to analyze the molecular characteristics and phenotypes of rare thalassemia gene variants, we used 434 cases with positive hematology screening as the cohort, then used SMRT technology and conventional gene diagnosis technology [(Gap-PCR, multiple ligation probe amplification technology (MLPA), PCR-reverse dot blot (RDB)] for thalassemia gene screening. Among the 434 enrolled cases, conventional technology identified 318 patients with variants (73.27%) and 116 patients without variants (26.73%), SMRT identified 361 patients with variants (83.18%), and 73 patients without variants (16.82%). The positive detection rate of SMRT was 9.91% higher than conventional technology. Combination of the two methods identified 485 positive alleles among 49 types of variant. The genotypes of 354 cases were concordant between the two methods, while 80 cases were discordant. Among the 80 cases, 76 cases had variants only identified in SMRT method, 3 cases had variants only identified in conventional method, and 1 false positive result by the traditional PCR detection technology. Except the three variants in HS40 and *HBG1-HBG2* loci, which was beyond the design of SMRT method in this study, all the other discordant variants identified by SMRT were validated by further Sanger sequencing or MLPA. The hematological phenotypic parameters of 80 discordant cases were also analyzed. SMRT technology increased the positive detection rate of thalassemia genes, and detected rare thalassemia cases with variable phenotypes, which had great significance for clinical thalassemia gene screening.

## Introduction

Thalassemia, also known as Mediterranean anemia, is a hereditary hemolytic anemia mainly caused by deletions or point mutations of globin genes. It is one of the most common single gene diseases in the world. The global thalassemia gene carriers comprise ~1.67% of the total population, which are mainly distributed in the Mediterranean coast, North Africa, the Middle East, the Indian mainland, Southeast Asia, and southern China [1]. Thalassemia is one of the most common genetic diseases in southern China. The pathogenic variants of thalassemia include single-nucleotide variations (SNVs), indels, and large fragments of copy number variants (CNVs) and structural variations (SVs). Among them, α-thalassemia is mainly caused by large fragment deletions, and β-thalassemia mainly involves point mutations. In China, simple and low cost red blood cell and hemoglobin tests are used as a first-tier screening strategy. Then molecular diagnosis will be performed for individuals with positive results of blood test. Conventional molecular diagnosis methods for detecting thalassemia genes include Gap-PCR, PCR-RDB, PCR-flow fluorescence hybridization, and MLPA. Other common technologies used in China include gene chip, Sanger sequencing, and next generation sequencing (NGS). Conventional screening methods can only detect a limited spectrum of gene mutations, which sometimes lead to misdiagnosis. NGS used in thalassemia screening can effectively reduce the need for various types of conventional genetic testing, but there could be missed diagnoses [2]. Although the probe hybridization target capture NGS method can simultaneously detect deletions and SNV/indels, the detection cost is high, and the accuracy is not ideal. Gap-PCR combined with NGS technology is currently used to compensate for the shortcomings of NGS capture sequencing technology. In addition, due to the high homology between *HBA2* and *HBA1* genes, the short-read NGS method cannot distinguish *HBA2* and *HBA1* effectively [3, 4]. With the advantage of long-molecule sequencing, PacBio real-time sequencing technology (SMRT) had been used for comprehensive and precious thalassemia test [5, 6]. In this study, SMRT technology and conventional methods were performed for 434 suspected carriers of thalassemia to simultaneously detect deletion and non-deletion variants of α-thalassemia and β-thalassemia. Compared to conventional methods, SMRT technology detected more abnormal hemoglobin variant sites on the *HBA1*, *HBA2*, and *HBB* genes, which illustrated the value of SMRT technology in the diagnosis of common and rare types of α-thalassemia and β-thalassemia variants.

## Patients and methods

### Patients

A total of 434 patients who attended Liuzhou Maternal and Child Health Hospital in Guangxi, China from January 2018 to December 2020, were included in the study. The enrolled patients should meet at least one of the following inclusion criteria: (1) routine hematology examination showed abnormal mean corpuscular volume (MCV ≤ 80 fL) and/or mean corpuscular hemoglobin (MCH ≤ 27 pg); (2) hemoglobin electrophoresis showed HbA2 < 2.5% or HbA2 ≥ 3.5% or elevated HbF or abnormal hemoglobin; (3) the results of conventional genetic diagnosis were inconsistent with the results of the hematology phenotype; (4) the patient gave birth to children with moderate or severe thalassemia; and (5) there may be abnormalities outside the scope of conventional genetic testing techniques. The exclusion criteria included: (1) incomplete basic clinical data; (2) the patient had other blood diseases; and (3) the patient had mental abnormalities or cognitive dysfunctions. The study group was comprised of 185 males and 249 females, with age range 3 days to 56 years, and an average age of 26.4 ± 12.59 years. This study was approval by the ethics committee of our hospital, and all research subjects or their legal guardians signed an informed consent form.

### Methods

#### Hematology and hemoglobin electrophoresis analysis

An automatic blood cell analyzer was used for routine blood analyses, and high-performance liquid chromatography was used for hemoglobin analysis to detect HbF, HbA2, HbH, and other hemoglobin variants.

#### Genomic DNA extraction

The magnetic bead method was used to extract nucleic acids (LabAid820; Xiamen Zhishan Biotechnology, Xiamen, China). The nucleic acid analyzer (ASP-2680; ACTGene, Piscataway, NJ, USA) was used to detect DNA concentration and purity. The A_260_/A_280_ of extracted DNA was between 1.6 and 1.9, and the concentration was 20–30 ng/µL.

#### α-thalassemia and β-thalassemia genotyping

Genomic DNA extracted from peripheral blood were used for thalassemia test. Gap-PCR (Yishengtang, Shenzhen, China) was performed for the four common α-thalassemia deletions [--^SEA^ (Southeast Asia), −α^3.7^ (rightward), −α^4.2^ (leftward) --^THAI^ (Thailand)] were performed using the gap-polymerase chain reaction (Gap-PCR). PCR-RDB assay (Yishengtang, Shenzhen, China) was performed for the three common non-deletional α-thalassemia mutations including Hb Constant Spring (Hb CS, *HBA2*: c.427T>C), Hb Quong Sze (Hb QS, *HBA2*: c.377T>C), and Hb Westmead (Hb WS, *HBA2*: c.369G>C), and the 17 known β-thalassemia mutations including −28 (A>G) (*HBB*: c.−76A>G), −29 (A>G) (*HBB*: c.−79A>G), −30 (T>C) (*HBB*: c.−80T>C), −32 (C>A) (*HBB*: c.−82C>A), codons 14/15 (+G) (*HBB*: c.45_46insG), codon 17 (A>T) (*HBB*: c.52A>T), codon 26 (or Hb E) (G>A) (*HBB*: c.79G>A), codons 27/28 (+C) (*HBB*: c.84_85insC), codon 31 (–C) (*HBB*: c.94delC), codons 41/42 (–TTCT) (*HBB*: c.126_129delCTTT), codon 43 (G>T) (*HBB*: c.130 G>T), codons 71/72 (+A) (*HBB*: c. 216_217insA), IVS-I-1 (G>T) (*HBB*: c.92+1G>T), IVS-I-5 (G>C) (*HBB*: c.92+5G>C), IVS-II-654 (C>T) (*HBB*: c.316-197C>T), CAP+1 (A>C) (*HBB*: c.−50A>C), and initiation codon (T>G) (*HBB*:c.2T>G). MLPA detection was performed using the P102 and P140 probe kit (MRC-Holland, Amsterdam, The Netherlands) to analyze the copy number variation of the deleted plutonium-Mediterranean gene. Capillary electrophoresis was performed on amplified products using the 3500Dx genetic Analyzer (Applied Biosystems, Foster City, CA, USA).

#### SMRT and data analysis

Genomic DNA was extracted from peripheral blood leukocytes using the QIAamp DNA blood mini kit (Qiagen, Hilden, Germany). Purified DNA samples were quantified using the Qubit dsDNA BR assay kit (Thermo Fisher Scientific, Waltham, MA, USA) using a Qubit 2.0 fluorometer (Life Technologies, Carlsbad, CA, USA). Samples were sent to an independent laboratory (Berry Genomics, Beijing, China) for sequencing using the Sequel II platform and data analysis (PacBio, Menlo Park, CA, USA). Briefly, genomic DNA samples were subjected to multiplex long-molecule PCR using optimized primers to generate specific amplicons that encapsulated currently known structural variation (SV) regions, single-nucleotide variations (SNVs), and indels (insertions and deletions) in the *HBA1*, *HBA2*, and *HBB* genes. After purification and end repair, the barcoded adapters were ligated to the 5′ and 3′ ends, and SMRT bell libraries were prepared using the sequel binding and internal Ctrl Kit 3.0 (PacBio). Primed DNA-polymerase complexes were loaded onto SMRT cells (PacBio) and sequencing was performed on the PacBio Sequel II system to generate 10–25 subjects per molecule. Following alignment of subreads, the consensus circular sequence was mapped to the GRCh38 reference and variants identified (FreeBayes software, version 1.2.0). Variant pathogenicity was classified according to general guidelines and from information provided in hemoglobin variant databases. Phenotypes were finally assigned from known genotypic–phenotypic associations. Large deletion variants was confirmed by Gap-PCR or MLPA. SNVs and indels were confirmed by PCR-RDB or Sanger sequencing.

### Sanger sequencing for *HBA* and *HBB* gene

Four sets of primer pairs were designed and used to amplify and sequence the α-globin genes (*HBA1* and *HBA2*) and β-globin gene (*HBB*): *HBA1*-F: 5′-TGG AGG GTG GAG ACG TCC TG-3′; *HBA1*-R: 5′-TCC ATC CTC TCC TCC CGC CCC TGC CTT TTC-3′; *HBA2*-F: 5′-TGG AGG GTG GAG ACG TCT TG-3′; *HBA2*-R: 5′-CCG TTG TTG GCA CAT TCC GG-3’;*HBB*-FP: 5′-AAC TCC TAA GCC AGT GCC AG-3′; AvaII-*HBB*-FP: 5′-TTG GGG ATC TGT CCA CTC CT-3′; AvaII-*HBB*-RP: 5′-CCA GCC TTA TCC CAA CCA TAA AAT AA-3′; and *HBB*-RP: 5′-ATG CAC TGA CCT CCC ACA TTC CCT-3′. DNA sequencing was performed using the Sanger dideoxy termination sequencing method, and the reference sequence was NM_000517. Amplification was performed using 50 ng of genomic DNA, and 20 pmol of forward (F) and reverse (R) primers, on a C1000 Thermal Cycler (Bio-Rad Laboratories, Hercules, CA, USA). The PCR products were sequenced on the ABI PRISM^®^ 3130 automated sequencer (Applied Biosystems).

### Data analysis

The subjects with positive test results were diagnosed as carriers or patients of thalassemia. The concordance of the two methods were then calculated. SPSS statistical software for Windows, version 22.0 (SPSS, Chicago, IL, USA) was used for statistical analyses. The measurement data are expressed as χ ± SD and the count data are expressed as examples and percentages.

## Results

### Comparison of genotyping results between traditional methods and SMRT technology

**Fig. 1:**
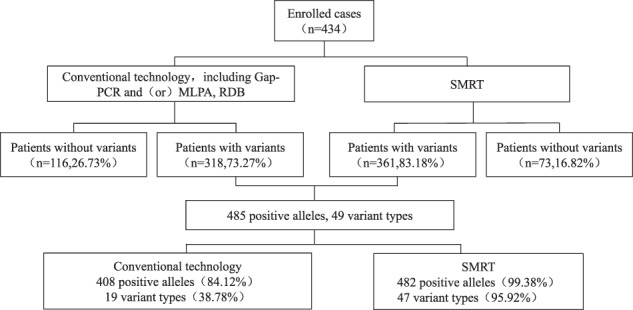
Combined analyses of the two techniques. Comparison of variants detection results between conventional technology and SMRT technology.

**Fig. 2:**
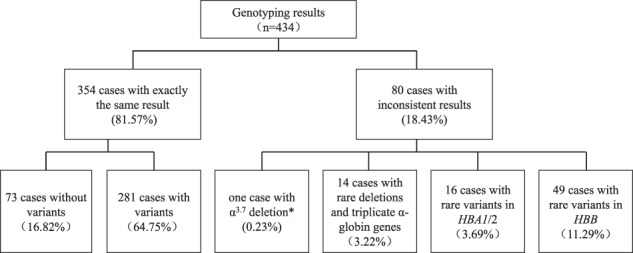
Comparison of genotyping results between conventional technology and SMRT technology *SMRT method showed that the genotype of sample D141966 was −α^3.7^/αα, while by conventional Gap-PCR it was −α^3.7^/−α^3.7^. Validation by MLPA confirmed D141966 had heterozygous −α^3.7^ deletion.

**Fig. 3:**
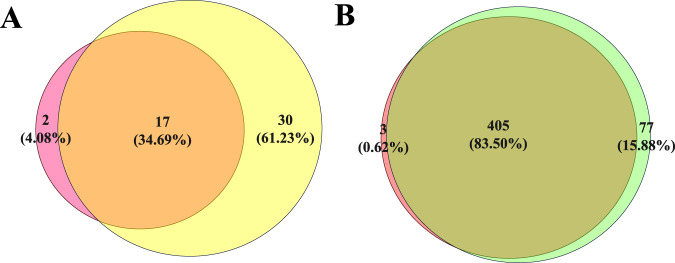
Distribution of 49 variant types and 485 positive alleles. **A** Distribution of 49 variant types. SMRT cannot detect but conventional techniques can detect (pink), SMRT and conventional techniques can detect (orange), SMRT can detect but conventional techniques failto detect (yellow). **B** Distribution of 485 positive alleles. SMRT cannot detect but conventional techniquescan detect (red), SMRT and conventional techniques can detect (yellow-green), SMRT can detect butconventional techniques fail to detect (light green).

**Fig. 4:**
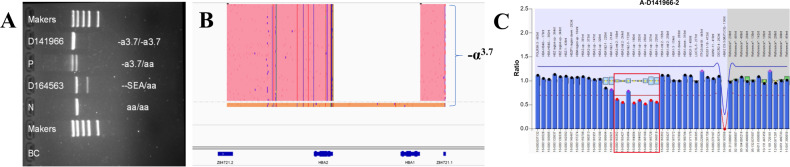
The genotyping results of sample D141966 by three technologies. **A** It was −α^3.7^/−α^3.7^ by Gap-PCR. **B** It was −α^3.7^/αα by SMRT. **C** It was −α^3.7^/αα by MLPA. Redboxed areas indicate the position of −α^3.7^ deletion.

**Fig. 5:**
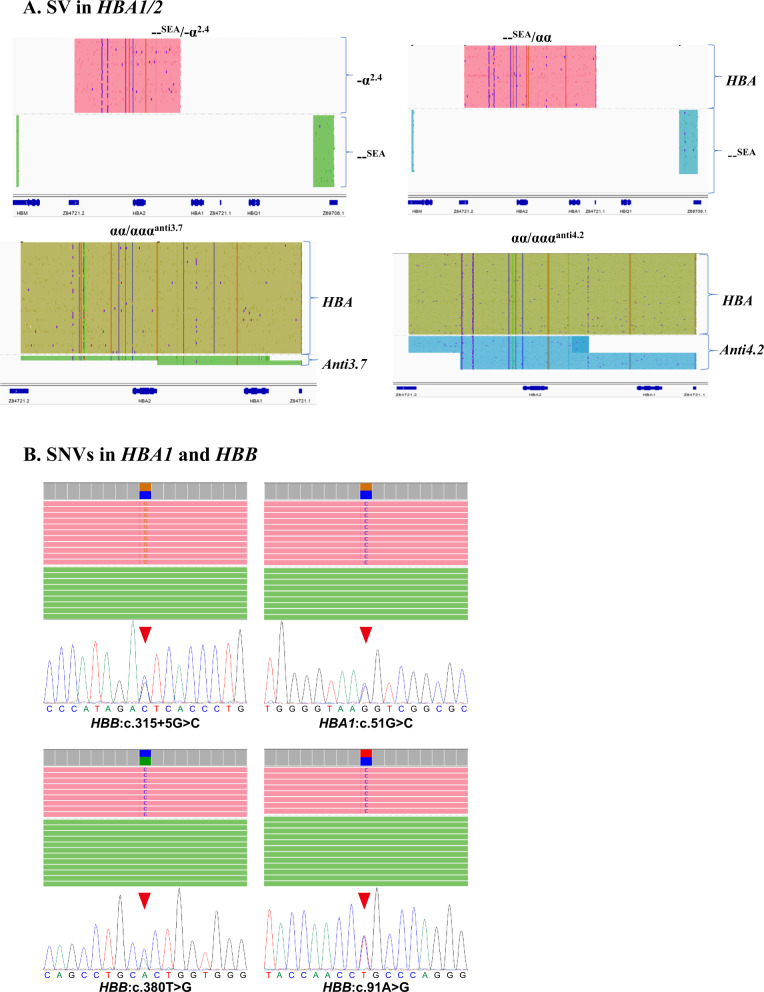
The Integrative Genomics Viewer plots of selected samples. Integrative Genomics Viewer plots of rare HBA1/2 structural variations (SVs; **A**) and single-nucleotide variations (SNVs; **B**) identified by comprehensive analysis of thalassemia alleles. Both the normal and variant alleles are visible in each profile. Red boxed areas indicate the position of the thalassemia variants.

Among the 434 enrolled cases, using conventional methods (gap-PCR and /or MLPA, RDB), 318 patients with variants (73.27%), and 116 patients without variants (26.73%) were identified. SMRT identified 361 patients with variants (83.18%) and 73 patients without variants (16.82%). The positive detection rate of SMRT was 9.91% higher than that of conventional methods. Combined analyses of the two techniques revealed that there were 485 positive alleles among 49 variant types (Fig. 1). Among them, conventional technology identified 408 positive alleles (84.12%) among 19 variant types (38.78%), and SMRT identified 482 positive alleles (99.38%) among 47 variant types (95.92%) (Fig. 2). Of the 49 types of variants, 30 types (61.23%) were detected by SMRT only, two types (4.08 %) were detected by conventional methods only, and 17 types (34.69%) were detected by both methods (Fig. 2). Of the 485 positive alleles, 77 (15.88%) were identified by SMRT only, three (0.62%) were identified by conventional methods and 405 (83.50%) were identified by both methods (Table 1 and Fig. 2). A total of 354 cases had completely concordant results between the two types of techniques, including 73 negative and 281 positive cases (Table 2 and Fig. 3), while 80 cases had discordant results (Fig. 3). Of the 80 cases, 14 patients had rare deletions and triplicate α-globin genes (Table 3), 16 patients had rare variants in the α-globin gene (Table 4), and 49 patients had rare variants in the β-globin gene (Table 5), one case had α^3.7^ deletion. The genotype of sample D141966 was −α^3.7^/αα by SMRT method, while it was −α^3.7^/−α^3.7^ by conventional Gap-PCR. Validated by MLPA technology, the genotype is the same as the result of SMRT technology (Fig. 4). The IGV plots of selected samples were displayed to show the thalassemia variants identified by SMRT (Fig. 5).

**Table 1:** Thalassemia variants identified by SMRT and conventional technology.

Common name	HGVS name	Clinical significance	Allele frequency^a^	Occurrence^a^	*n* (%)	Detection range of conventional technology	Detection range of SMRT	Verification method	Reference
-α^4.2^	N/A1	Pathogenic	None	Chinese	130 (26.80)	Yes	Yes	Gap-PCR/MLPA	[2]
				East Asian					
				Indian					
-α3.7	NG_000006.1:g.34164_37967del3804	Pathogenic	None	African, Far East, Indian, Mediterranean	95 (19.59)	Yes	Yes	Gap-PCR/MLPA	[2]
--^SEA^	NG_000006.1:g.26264_45564del19301	Pathogenic	None	East Asian	94 (19.38)	Yes	Yes	Gap-PCR/MLPA	[2]
--^THAI^	NC_000016.10:g.149863_183312del	Pathogenic	None	Thai	2 (0.41)	Yes	Yes	Gap-PCR/MLPA	[2]
-α^2.4^	N/A1	Pathogenic	None	Chinese	1 (0.21)	No	Yes	Gap-PCR/MLPA	[8]
*HS-40* deletion	NC_000016.10:g.(47217_113592)_(113687_143639)del	Pathogenic	None	Chinese	2 (0.41)	Yes	No	MLPA	[9]
*HBG1-HBG2*	N/A1	Uncertain-significance	None	Chinese	1 (0.21)	Yes	No	MLPA	This study
Hkαα	N/A1	Uncertain-significance	None	Chinese	6 (1.24)	No	Yes	Gap-PCR	[12]
ααα^anti-4.2^	N/A1	Uncertain-significance	None	Chinese	2 (0.41)	No	Yes	Gap-PCR	[10, 11]
ααα^anti-3.7^	N/A1	Uncertain-significance	None	Chinese	2 (0.41)	No	Yes	Gap-PCR	[10, 11]
CD142 (TAA>CAA)	*HBA2:c.427T*>*C*	Pathogenic	14/248854, GnomAD_exome	Arabian	18 (3.71)	Yes	Yes	RDB/Sanger	[2]
				Cambodian					
				Chinese					
				Greek					
				Indian					
				Indonesian					
				Laotian					
				Malasian					
				Sicilian					
				Vietnamese					
CD125 (CTG>CCG)	*HBA2:c.377T*>*C*	Pathogenic	1/136864, GnomAD	Chinese	3 (0.62)	Yes	Yes	RDB/Sanger	[2]
CD122 (CAC>CAG)	*HBA2:c.369C*>*G*	Uncertain-significance	19/116218, ExAC	Chinese	2 (0.41)	Yes	Yes	RDB/Sanger	[2]
				Laotian					
CD11 (AAG>CAG)	HBA1:c.34A>C	Uncertain-significance	19/116218, ExAC	Chinese	1 (0.21)	No	Yes	Sanger	[13]
CD16 (AAG>AAC)	*HBA1:c.51G*>*C*	Uncertain-significance	None	Chinese	1 (0.21)	No	Yes	Sanger	[14]
				Pakistani					
CD27 (AAG>AAT)	*HBA1:*c.84G>T	Uncertain-significance	None	Chinese	2 (0.41)	No	Yes	Sanger	[15]
CD6 (GAC>TAC)	*HBA1:*c.19G>T	Uncertain-significance	None	Vietnamese	1 (0.21)	No	Yes	Sanger	[16]
CD18 (GGC>CGC)	*HBA1:*c.55G>C	Uncertain-significance	2/138328, GnomAD	Chinese	4 (0.82)	No	Yes	Sanger	[17]
				Indian					
				Saudi Arabian					
Init CD (ATG>A-G)	*HBA2:*c.2delT	Pathogenic	None	Vietnamese	1 (0.21)	No	Yes	Sanger	[18]
Init CD (ATG>ACG)	*HBA2*:c.2T>C	Pathogenic	0/654, ALFA	Italian	1 (0.21)	No	Yes	Sanger	[19]
CD17 (GTC>TTC)	*HBA2:*c.52G>T	Uncertain-significance	None	Chinese	1 (0.21)	No	Yes	Sanger	[20]
CD30 (GAG>CAG)	*HBA2:*c.91G>C	Uncertain-significance	1/264690, TOPMED	Chinese	3 (0.62)	No	Yes	Sanger	[21]
−22 C>T	*HBA2:*c.−59C>T	Pathogenic-likely-pathogenic	None	Nedlands	1 (0.21)	No	Yes	Sanger	[22]
CD85 (GAC>AAC)	*HBA2:c.256G*>*A*	Uncertain-significance	None	English	1 (0.21)	No	Yes	Sanger	[23]
CD41/42 (-TTCT)	*HBB:*c.126_129delCTTT	Pathogenic	7/140174, GnomAD	Chinese 41.84%	22 (4.54)	Yes	Yes	RDB/Sanger	[2]
				English 4.35%					
				Indonesian 1.69%					
				Japanese 5.99%					
				Korean 4.17%					
				Malaysian 26.32%					
				Pakistani 6.7%					
				Punjabi 13.22%					
				Singapore 37.59%					
				Taiwanese 30.63%					
				Thai 37.24%					
CD17 (AAG>TAG)	*HBB:*c.52A>T	Pathogenic	3/140268, GnomAD	Chinese 14.1%	15 (3.09)	Yes	Yes	RDB/Sanger	[2]
				Indonesian 1.69%					
				Japanese 0.32%					
				Korean 16.67%					
				Malaysian 5.26%					
				Singapore 9.02%					
				Taiwanese 8.13%					
				Thai 18.56%					
−28 (A>G)	*HBB:*c.−78A>G	Pathogenic-likely-pathogenic	1/140226, GnomAD	Chinese 12.31%	10 (2.06)	Yes	Yes	RDB/Sanger	[2]
				Japanese 0.32%					
				Malaysian 6.43%					
				Taiwanese 9.38%					
				Thai 6.83%					
CD26 (GAG>AAG)	*HBB:*c.79G>A	Pathogenic	9/140272, GnomAD	Thai 0.12%	5 (1.03)	Yes	Yes	RDB/Sanger	[2]
CD71/72(+A)	*HBB:*c.216_217insA	Pathogenic	2/251430, GnomAD_exome	Chinese	3 (0.62)	Yes	Yes	RDB/Sanger	[2]
				East Asian					
IVS-II-654 (C>T)	*HBB:*c.316-197C>T	Pathogenic	7/140170, GnomAD	Chinese 21.37%	2 (0.41)	Yes	Yes	RDB/Sanger	[2]
				Indonesian 11.86%					
				Japanese 11.99%					
				Malaysian 10.53%					
				Russian 1.52%					
				Singapore 25.56%					
				Taiwanese 46.25%					
				Thai 5.13%					
CD14/15(+G)	HBB:c.45dupG	Pathogenic	1/251224, GnomAD_exome	Chinese	1 (0.21)	Yes	Yes	RDB/Sanger	[2]
				Thai 0.12%					
−29 (A>G)	*HBB:*c.−79A>G	Pathogenic	127/140260, GnomAD	Algerian 3.8%	1 (0.21)	Yes	Yes	RDB/Sanger	[2]
				Black 59.38%					
				Chinese 2.37%					
				Malaysian 0.58%					
				Taiwanese 0.63%					
CD27/28(+C)	*HBB:*c.84_85insC	Pathogenic	1/264690, TOPMED	Chinese 0.59%	1 (0.21)	Yes	Yes	RDB/Sanger	[2]
				Singapore 0.75%					
				Taiwanese 2.5%					
				Thai 0.24%					
IVS-I-5 (G>C)	*HBB:*c.92+5G>C	Pathogenic	1/140258, GnomAD	Frequent in Asian Indian, UAE, and East Asian populations	1 (0.21)	Yes	Yes	RDB/Sanger	[2]
−50 (G>A)	*HBB:*c.−100G>A	Uncertain-significance	2/140260, GnomAD	Chinese	1 (0.21)	No	Yes	Sanger	[24]
−86 (C>G)	*HBB:*c.−136C>G	Pathogenic	0/78698, GnomAD	Lebanese,	1 (0.21)	No	Yes	Sanger	[25]
				Thai 0.24%					
IVS-II-5 (G>C)	*HBB:*c.315+5G>C	Pathogenic	1/140204, GnomAD	Chinese 0.15%	10 (2.06)	No	Yes	Sanger	[26]
CD126 (GTG>GGG)	*HBB:*c.380T>G	Pathogenic	1/140228, GnomAD	German	1 (0.21)	No	Yes	Sanger	[27]
				Italian					
				Thai					
−31 (A>C)	*HBB:*c.−81A>C	Pathogenic	1/264690, TOPMED	Italian	1 (0.21)	No	Yes	Sanger	[22]
				Chinese					
CD30 (A>G)	*HBB:*c.91A>G	Likely-pathogenic	None	Sephardic Jewish	5 (1.03)	No	Yes	Sanger	[28]
CD56 (GGC>GAC)	*HBB:*c.170G>A	Likely-benign	5/251448, GnomAD_exome	Found in Thai, Indonesian, Black, and Chinese families	5 (1.03)	No	Yes	Sanger	[29]
CD 64 (GGC>AGC)	*HBB:*c.193G>A	Likely-benign	None	Chinese	1 (0.21)	No	Yes	Sanger	[30]
CD77 (CAC>TAC)	*HBB:*c.232C>T	Likely-benign	1/251428, GnomAD_exom	Caucasian	1 (0.21)	No	Yes	Sanger	[31]
				Indonesian					
				Japanese					
				Swedish					
−198A>G	*HBB:*c.−248A>G	Uncertain-significance	None	This study	3 (0.62)	No	Yes	Sanger	This study
CD113 (GTG>GAG)	*HBB:*c.341T>A	Pathogenic	2/140270, GnomAD	American	14 (2.89)	No	Yes	Sanger	[32]
				Chinese					
CD143 (CAC>CGC)	*HBB:*c.431A>G	Likely-pathogenic	1/251362, GnomAD_exome	American	1 (0.21)	No	Yes	Sanger	[33]
				Italian					
IVS- II-806 (G>C)	*HBB:*c.316-45G>C	Benign-likely-benign	34/140174, GnomAD	Chinese	3 (0.62)	No	Yes	Sanger	This study
IVS- II-672 (A>C)	*HBB:*c.316-179A>C	Benign-likely-benign	None	Chinese	1 (0.21)	No	Yes	Sanger	This study
IVS -II-308 (-A)	*HBB:*c.315+308delA	Benign-likely-benign	None	Chinese	1 (0.21)	No	Yes	Sanger	This study
Total	–	–	–	–	485 (100)	–	–	–	–

*Gap-PCR* Gap- polymerase chain reaction, *MLPA* multiple ligation probe amplification technology, *RDB* reverse dot blot.

^a^Data come from HbVar (https://globin.bx.psu.edu/hbvar/hbvar.html), genomAD (https://gnomad.broadinstitute.org/), and dbSNP (https://www.ncbi.nlm.nih.gov/snp/).

**Table 2:** Genotype analysis with exactly the same test results for the two types of technologies.

SMRT	Conventional technologies	Concordance	*n*
-α^4.2^/αα	-α^4.2^/αα	Yes	104
-α^3.7^/αα	-α^3.7^/αα	Yes	43
--^SEA^/-α^3.7^	--^SEA^/-α^3.7^	Yes	32
--^SEA^/αα	--^SEA^/αα	Yes	24
--^SEA^/-α^4.2^	--^SEA^/-α^4.2^	Yes	13
α^CS^α/αα	α^CS^α/αα	Yes	6
-α^3.7^/α^CS^α	-α^3.7^/α^CS^α	Yes	4
-α^3.7^/-α^4.2^	-α^3.7^/-α^4.2^	Yes	2
-α^4.2^/α^CS^α	-α^4.2^/α^CS^α	Yes	2
α^WS^α/αα	α^WS^α/αα	Yes	2
-α^4.2^/-α^4.2^	-α^4.2^/-α^4.2^	Yes	1
--^SEA^/-α^4.2^	--^SEA^/-α^4.2^	Yes	1
--^THAI^/α^CS^α	--^THAI^/α^CS^α	Yes	1
α^CS^α/α^QS^α	α^CS^α/α^QS^α	Yes	1
α^QS^α/αα	α^QS^α/αα	Yes	1
β^CD17(AAG>TAG)^/β^N^	β^CD17(AAG>TAG)^/β^N^	Yes	9
β^CD41/42(−TTCT)^/β^N^	β^CD41/42(−TTCT)^/β^N^	Yes	9
β^CD41/42(−TTCT)^/β^CD17(AAG>TAG)^	β^CD41/42(−TTCT)^/β^CD17(AAG>TAG)^	Yes	2
β^CD71/72(+A)^/β^N^	β^CD71/72(+A)^/β^N^	Yes	2
β^CD26(GAG>AAG)^/β^N^	β^CD26(GAG>AAG)^/β^N^	Yes	2
β^−28(A>G)^/β^−28(A>G)^	β^−28(A>G)^/β^−28(A>G)^	Yes	1
β^CD14/15(+G)^/β^N^	β^CD14/15(+G)^/β^N^	Yes	1
β^CD41/42(−TTCT)^/β^−28(A>G)^	β^CD41/42(−TTCT)^/β^−28(A>G)^	Yes	1
β^IVS-I-5(G>C)^/β^N^	β^IVS-I-5(G>C)^/β^N^	Yes	1
β^IVS-II-654(C>T)^/β^N^	β^IVS-II-654(C>T)^/β^N^	Yes	1
β^IVS-II-654(C>T)^/β^−28(A>G)^	β^IVS-II-654(C>T)^/β^−28(A>G)^	Yes	1
β^−29(A>G)^/β^N^	β^−29(A>G)^/β^N^	Yes	1
β^−28(A>G)^/β^N^	β^−28(A>G)^/β^N^	Yes	1
α^CS^α/αα with β^CD17(AAG>TAG)^/β^−28(A>G)^	α^CS^α/αα with β^CD17(AAG>TAG)^/β^−28(A>G)^	Yes	1
--^SEA^/αα with β^CD41/42(−TTCT)^/β^−28(A>G)^	--^SEA^/αα with β^CD41/42(−TTCT)^/β^−28(A>G)^	Yes	2
α^QS^α/αα with β^CD17(AAG>TAG)^/β^N^	α^QS^α/αα with β^CD17(AAG>TAG)^/β^N^	Yes	1
--^SEA^/αα with β^−28(A>G)^/β^−28(A>G)^	--^SEA^/αα with β^−28(A>G)^/β^−28(A>G)^	Yes	1
--^SEA^/αα with β^CD41/42(−TTCT)^/β^CD41/42(−TTCT)^	--^SEA^/αα with β^CD41/42(−TTCT)^/β^CD41/42(-TTCT)^	Yes	1
--^SEA^/-α^3.7^ with β^CD71/72(+A)^ /β^CD41/42(−TTCT)^	--^SEA^/-α^3.7^ with β^CD71/72(+A)^ /β^CD41/42(-TTCT)^	Yes	1
--^SEA^/-α^3.7^ with β^CD17(AAG>TAG)^/β^N^	--^SEA^/-α^3.7^ with β^CD17(AAG>TAG)^/β^N^	Yes	1
--^SEA^/-α^4.2^ with β^CD41/42(−TTCT)^/β^CD26(GAG>AAG)^	--^SEA^/-α^4.2^ with β^CD41/42(−TTCT)^/β^CD26(GAG>AAG)^	Yes	1
-α^3.7^/α^CS^α with β^CD26(GAG>AAG)^/β^N^	-α^3.7^/α^CS^α with β^CD26(GAG>AAG)^/β^N^	Yes	1
-α^4.2^/αα with β^CD41/42(−TTCT)^/β^CD17(AAG>TAG)^	-α^4.2^/αα with β^CD41/42(−TTCT)^/β^CD17(AAG>TAG)^	Yes	1
-α^4.2^/αα with β^CD26(GAG>AAG)^/β^N^	-α^4.2^/αα with β^CD26(GAG>AAG)^/β^N^	Yes	1
Total	–	–	281

**Table 3:** Hematology examination and hemoglobin electrophoresis results of 14 cases with rare deletions and triplicate α-globin genes.

Genotype	*n*	Age (y)	RBC	Hb (g/L)	MCV (fL)	MCH (pg)	HbA (%)	HbA2 (%)	HbF (%)	HbH (%)
--^SEA^/-α^2.4^	1	39	4.05	85	69.3	21.2	68.2	1.2	0.4	20.1
*HS-40* deletion/αα with β^CD41/42(−TTCT)^/β^N^	1	0.1	5.03	127	79.1	25.2	10.6	0.2	86.6	–
-α^3.7^/*HS-40* deletion	1	44	4.78	84	57.3	17.5	80.2	2.1	0.4	5.3
αα/ααα^anti3.7^	1	34	4.97	150	92	30.1	76.5	2.1	12.3	–
αα/ααα^anti3.7^ with β^CD27/28(+C)^ /β^N^	1	36	4.45	102	71.2	23	69.7	3.7	19.5	–
αα/ααα^anti4.2^	1	37	4.67	143	91.5	29.1	81.6	2	16.2	–
αα/ααα^anti4.2^ with β^CD41/42(−TTCT)^/β^N^	1	50	4.41	102	77.7	23.2	85.3	4.8	9.9	–
*HBG1-HBG2* deletion with β^CD41/42(−TTCT)^/β^N^	1	3d	4.75	165	103	34.8	15	0	85	–
--^SEA^/Hkαα	4	32.00 ± 9.31	5.75 ± 0.56	130.50 ± 12.15	69.80 ± 2.19	22.70 ± 1.16	94.70 ± 6.33	2.25 ± 0.26	0.08 ± 0.15	–
HKαα/αα	2	35.00 ± 1.41	4.34 ± 0.54	134.50 ± 3.54	94.80 ± 9.48	31.15 ± 3.18	90.60 ± 9.33	3.00 ± 0.28	2.05 ± 2.90	–
Total	14	–	–	–	–	–	–	–	–	–

**Table 4:** Hematology examination and hemoglobin electrophoresis results of 16 cases with rare variants in *HBA1/2* gene.

Genotype	Cases (*n*)	Age (y)	RBC	Hb (g/L)	MCV (fL)	MCH (pg)	HbA (%)	HbA2 (%)	HbF (%)	HbH (%)	Abnormal hemoglobin
--^SEA^/*HBA2:c.2delT*	1	31	4.71	81	61.6	17.1	61.2	1.1	0.4	31	–
--^SEA^/*HBA2:c.2T*>*C*	1	1	4.92	81	65.4	19.2	89.3	0.6	2.2	8.1	–
--^SEA^/*HBA2:c.52G*>*T*	1	33	5.65	106	63.2	18.7	89	0.8	0	8.9	–
-α^3.7^/*HBA1:c.19G*>*T*	1	28	5.64	150	80.3	26.6	73.2	2.2	0.5	–	4.129 min
*HBA1:c.34A*>*C*	1	31	4.93	165	97.5	33.5	75.7	1.9	0	–	22.4%
*HBA1:c.51G*>*C*	1	0.5	3.31	106	99.8	31.9	62.8	2	13.7	–	17.2 min
*HBA1:c.55G*>*C*	2	21.10 ± 29.56	5.10 ± 0.16	127.00 ± 33.94	76.15 ± 15.06	24.85 ± 5.73	73.45 ± 16.33	2.2	11.85 ± 16.76	–	4.498 min
--^SEA^/*HBA1:c.55G*>*C*	2	33.00 ± 4.24	5.40 ± 0.71	105.50 ± 23.33	63.55 ± 5.59	19.35 ± 1.77	64.75 ± 0.49	2.85 ± 0.07	0.65 ± 0.35	–	4.515–4.528 min
--^SEA^/*HBA1:c.84G*>*T*	2	30.00 ± 2.83	5.43 ± 0.34	123.00 ± 14.14	72.75 ± 4.17	22.65 ± 1.06	71.10 ± 22.20	2.05 ± 0.21	0.75 ± 0.07	–	−/2.310 min
*HBA2:c.91G*>*C*	2	41.00 ± 4.24	4.62 ± 0.66	144.50 ± 20.51	92.20 ± 0.42	31.25 ± 0.07	74.20 ± 0.14	1.95 ± 0.35	0	–	23.5%
*HBA2:c.−59C*>*T*+*HBA2:c.91G*>*C*	1	27	5	137	83.4	27.4	68.3	21.6	0.4	–	3.784/4.349 min
*HBA2:c.256G*>*A*	1	0.5	4.54	126	84	27.8	64.5	1.6	3.1	–	4.059 min
Total	16	–	–	–	–	–	–	–	–	–	–

Abnormal hemoglobin [peak time(min) or proportion (%)].

**Table 5:** Hematology examination and hemoglobin electrophoresis results of 49 patients with rare variants in *HBB*.

Genotype	Cases (*n*)	Age (y)	RBC	Hb (g/L)	MCV (fL)	MCH (pg)	HbA (%)	HbA2 (%)	HbF (%)	HbH (%)	Abnormal hemoglobin
*HBB*:c.315+5G>C/WT	6	32.17 ± 5.74	5.16 ± 0.41	143.5 ± 12.44	84.42 ± 3.81	27.85 ± 1.28	86.50 ± 4.86	3.68 ± 0.10	0.67 ± 0.45	–	–
*HBB*:c.315+5G>C /c.52A>T	1	8	3.75	94	76.5	25	70	3.6	17.3	–	–
--^SEA^/αα with *HBB*:c.315+5G>C/c.315+5G>C	1	31	4.23	101	73.6	23.7	94.3	4.5	1.2	–	–
-α^4.2^/αα with *HBB*:c.315+5G>C/WT	1	23	3.82	91	76.5	23.9	85.9	3.6	0.3	–	–
*HBB*:c.−81A>C/WT	1	33	5.8	137	78.8	23.4	83.2	4.5	1.2	–	–
*HBB*:c.91A>G/WT	5	19.00 ± 13.67	5.66 ± 0.58	114.20 ± 11.34	63.18 ± 3.45	20.24 ± 1.05	89.34 ± 6.42	5.20 ± 0.32	1.04 ± 0.68	–	–
*HBB*:c.−100G>A/WT	1	41	4.63	122	80.6	26.4	83.7	3.9	1.9	–	–
--^THAI^/αα with *HBB*:c.193G>A / WT	1	27	6.28	139	70.8	22.2	85.6	3.7	0.3	–	–
*HBB*:c.−136C>G /WT	1	26	5.57	142	78.9	25.5	90.4	5.3	4.3	–	–
*HBB*:c.380T>G/WT	1	31	4.98	123	78.6	24.7	96	3.7	0.3	–	–
*HBB*:c.170G>A/WT	5	17.32 ± 15.86	4.19 ± 0.66	127.60 ± 20.46	91.64 ± 7.99	30.52 ± 2.49	39.74 ± 10.89	2.24 ± 0.81	16.04 ± 26.43	–	2.066–2.497 min
*HBB*:c.232C>T/WT	1	33	4.45	136	93.1	30.5	47	2.7	1.7	–	1.802 min
*HBB*:c.341T>A/WT	11	23.82 ± 17.19	4.46 ± 0.47	126.18 ± 17.66	86.39 ± 7.26	28.28 ± 2.42	54.54 ± 10.70	2.80 ± 0.35	2.24 ± 5.61	–	45%
*HBB*:c.341T>A/c.315+5G>C	1	28	4.82	103	68.4	21.5	0	4.6	1.9	–	93.5%
--^SEA^/αα with *HBB*:c.341T>A/ WT	1	30	6.28	137	68.5	21.8	61	3.2	0	–	35.8%。
α^CS^α/ αα with *HBB*:c.341T>A/WT	1	29	4.55	116	77.9	25.4	57.1	2.7	0.3	–	40%
*HBB*:c.431A>G/WT	1	38	5.18	152	89.2	29.3	49.8	46.5	0.4	–	3.48 min
*HBB*:c.−248A>G/WT	1	31	4.98	148	88.6	29.7	86	1.8	0.2	–	4.742 min
-α^3.7^/ α^CS^α with *HBB*:c.−248A>G/WT	1	24	3.76	88	76.1	23.3	97.1	1.8	0	–	1.1% /Hb CS?
--^SEA^/αα with *HBB*:c.−248A>G/WT	1	33	5.08	109	65.6	21.5	87.4	1.5	1	–	–
-α^3.7^/αα with *HBB:*c.316-45G>C/WT	2	33.25 ± 6.09	5.12 ± 0.75	131.88 ± 24.74	79.40 ± 8.93	25.74 ± 3.25	76.39 ± 17.56	7.90 ± 15.61	0.33 ± 0.32	–	–
--^SEA^/-a^3.7^ with *HBB*:c.316-45G>C/WT	1	40	5.09	100	65.7	19.6	76.7	1.6	0.1	16.6	–
-α^4.2^/αα with *HBB*:c.316-45G>C/WT	1	40	4.51	107	70.7	23.8	98	2	0	–	–
-α^3.7^/αα with *HBB*:c.316-179A>C/WT	1	33	4.42	129	88.4	29.1	96.3	2.8	0.9	–	–
-α^4.2^/αα with *HBB*:c.315+308delA/WT	1	30	5.92	161	79.1	27.1	69.1	1.7	0	–	28.6%/Hb Q-Thailand?
Total	49	–	–	–	–	–	–	–	–	–	–

Abnormal hemoglobin [Peak time(min) or proportion (%)].

### Hematology examination and hemoglobin electrophoresis results in patients with rare deletions and triplicate α-globin genes

A total of 14 rare cases of α and β-globin gene deletions, or triplicate α-globin genes were found (Table 3). Among them, the result of hemoglobin electrophoresis of patients with --^SEA^/−α^2.4^ and −α^3.7^/HS-40 deletion showed the presence of HBH peak. The routine hematology examination results of patients with αα/ααα^anti3.7^ and αα/ααα^anti4.2^ were normal, but HbA2 was decreased and HbF was significantly increased to 12.3% and 16.2%, respectively. When αα/ααα^anti3.7^ and αα/ααα^anti4.2^ were combined with β-thalassemia, they were assigned as intermediate β-thalassemia, and HbF was also significantly increased. Patients with HKαα/αα presented with silent α-thalassemia, and patients with HKαα/--^SEA^ presented with mild α-thalassemia. The *HBG1*-*HBG2* deletion combined with β^CD41/42(-TTCT)^/β^N^ was clinically manifested as mild β-thalassemia (Table 3).

### Hematology examination and hemoglobin electrophoresis results in patients with rare variants in *HBA1/2*

Sixteen rare *HBA1/2* variants were found, including --^SEA^/*HBA2*:c.2delT, --^SEA^/*HBA2*:c.2T>C,--^SEA^/*HBA2*:c.52G>T,-α^3.7^/*HBA1*:c.19G>T,*HBA1*:c.34A>C,*HBA1*:c.51G>C,*HBA1*:c.55G>C,--^SEA^/*HBA1*:c.55G>C,--^SEA^/*HBA1*:c.84G>T,*HBA2*:c.91G>C,*HBA2*:c.−59C>T combined with *HBA2*:c.91G>C, *HBA2*:c.256G>A. Among them, *HBA1: c.34A*>*C* and *HBA1: c.51G*>*C* had normal routine blood test results, but abnormal hemoglobin was detected. The routine blood phenotypes of the genotypes *HBA1: c.84G*>*T*, *HBA1:c.19G*>*T*, and *HBA1:c.55G*>*C* were normal. If it was compounded with other deletion variant, it manifested as mild or silent thalassemia, and abnormal hemoglobin was detected. The --^SEA^/*HBA2*:c.2delT, --^SEA^/*HBA2*: c.2T>C, and --^SEA^/*HBA2*: c.52G>T manifested as non-deletion HbH disease. *HBA2*: c.91G>C had normal hematology phenotype, though with abnormal hemoglobin, and reduced HbA2. In cases with *HBA2*: c.−59C>T/*HBA2*: c.91G>C compound heterozygous variants, hemoglobin electrophoresis showed abnormal hemoglobin peaks at 3.784 and 4.349 min, the routine hematology phenotype was normal, and HbA2 was significantly increased. *HBA2*: c.256G>A cases had a normal phenotype, though abnormal hemoglobin was detected, and HbA2 was reduced (Table 4).

### Hematology examination and hemoglobin electrophoresis results in patients with rare variants in *HBB*

The c.−100G>A, c.−136C>G, c.315+5G>C, c.380T>G, and c.−81A>C belonged to beta+ thalassemia (partial loss of function of β-globin gene). Heterozygous variants of these types showed silent or mild β-thalassemia. c.91A>G belonged to beta0 thalassemia (complete loss of function of the β-globin gene), and heterozygous mutations manifested as mild β thalassemia. The hematological phenotype of c.170G>A, c.431A>G, c.232C>T, c.341T>A, and c.431A>G heterozygous variation was normal, the HbA2 and HbF contents were within the reference range, but abnormal hemoglobins were detected. Abnormal hemoglobin of case with c.341T>A/c.315+5G>C accounted for 93.5%, and HbA was almost undetectable. HbA2 content of case with c.431A>G heterozygous variation was increased. The hematological phenotype of c.−248A>G heterozygous variation was normal, and mainly manifested as decreased HbA2 content. c.316-45G>C, c.316-179A>C, and c.315+308delA combined with other types of α-thalassemia were manifested as silent or mild α-thalassemia (Table 5).

## Discussion

Thalassemia is a single gene disease, which is difficult to cure but more straightforward to diagnose and be prevented clinically. Its gene mutation types are diverse and complex. As of 2021, the LOVD (https://databases.lovd.nl/shared/genes) database had more than 2000 thalassemia and abnormal hemoglobin-related variant sites, and most of the sites have not been studied by conventional genetic testing methods, especially the large deletion variant type. At present, common clinical testing techniques for thalassemia genes include Gap-PCR, reverse dot hybridization, PCR-flow fluorescence hybridization, gene chip, MLPA, Sanger sequencing, and next generation sequencing. The conventional screening mode can only detect variants in known gene loci, which is far from sufficient for the detection of other variant loci, leading to missed diagnoses and misdiagnoses. There is therefore an urgent need to use more accurate and effective diagnostic techniques to screen thalassemia patients in clinical practice. In recent years, there have been reports of missed detections of thalassemia using conventional genetic testing methods. The SMRT technology can detect the thalassemia gene without interrupting the DNA, and can directly read the full-length gene sequence. The DNA does not need to be amplified by PCR during sequencing, which facilitates individual sequencing of each DNA molecule, and it has very long read lengths (a read length up to 30–100 kb), high accuracy (QV30 > 99.8%), no GC preference, and single-molecule resolution characteristics [7]. SMRT technology can facilitate the simultaneous detection of α-thalassemia and β-thalassemia in 1 μL of whole blood or 10–15 mL of amniotic fluid sample. It can also detect hotspots and rare variant sites and their arrangements with high accuracy, including comprehensive coverage of 2062 variant sites related to thalassemia, and detection of 18 α-globin gene deletion variants, four α-globin genes triplicate and two β-globin gene deletion. It can detect 96 samples at a time with high efficiency and high accuracy.

Xu et al. [5] first used the SMRT to sequence full-length thalassemia-related genes (*HBA1/2* and *HBB*) to obtain complete variant information of two alleles that were difficult to obtain by conventional genetic testing techniques. Twelve hospitals in southern China assessed a comprehensive analysis of thalassemia alleles (CATSA) for identifying both α and β thalassemia genetic carrier status by third-generation sequencing (TGS). Compared with standard thalassemia variant PCR panel testing, TCS can detected 33 more positive variants, and found that the traditional PCR detection technology had 1 false negative and 8 false positive result [6]. The present study used the SMRT and conventional technologies to test the thalassemia gene in the thalassemia screening positive population in this area. The results showed that the percentage of thalassemia gene was high and the genotype was complex, rare variant types of thalassemia and the phenotypes were diverse. Among the 434 cases, 49 variant types were detected, of which 19 were detected by conventional technology and 47 were detected by SMRT technology. Compared with conventional technology, SMRT technology detected 28 more variant types. The positive detection of SMRT was 9.91% higher than that of conventional technology, and SMRT technology increased the detection of thalassemia genes. At present, the detection range of the reagents we used only included 2062 variant sites related to thalassemia on the *HBA1/2* and *HBB* genes. HS-40 deletion occurs upstream of the α-globin gene cluster, and *HBG1-HBG2* deletion occurs upstream of the *HBB* gene cluster. The SMRT method developed in this study focused on detection of variants in *HBA1*, *HBA2*, and *HBB* genes, which consisted the vast majority of thalassemia variants. With expanded primer pairs, the SMRT technology can definitely detect HS-40 and *HBG1-HBG2* deletions. However, the sequencing cost will increase with more primer pairs [7]. So, it was the limit of the design of SMRT method in this study but not SMRT technology itself.

This study found 14 cases of rare deletions or triplicate α-globin genes. Among them, the --^SEA^/−α^2.4^ and −α^3.7^/HS-40 deletion patients all manifested with HbH disease [8, 9]. Carriers of ααα^anti3.7^ and ααα^anti-4.2^ had normal phenotypes, but HbF was significantly increased by 12.3% and 16.2%, respectively, and HbA2 was reduced. When compounded with β-thalassemia, it can manifest as intermediate β-thalassemia due to the aggravation of the imbalance between the α and β chains, and HbF is also significantly increased [10, 11]. In the present study, among the thalassemia carriers whose detection results were −α^3.7^/αα by conventional methods, two of them were found to be HKαα/αα using SMRT technology, and the misdiagnosis rate was as high as 4.17% (2/48). HKαα/αα patients presented with silent α-thalassemia, and HKαα/--^SEA^ patients presented with mild α-thalassemia, which is consistent with past reports [12]. Although the *HBG1-HBG2* deletion combined with c.126_129delCTTT/WT had two allelic variants in the *HBB* gene, *HBG1-HBG2* was functionally closed in adulthood and did not affect the expression of β globin, so it was clinically mild β-thalassemia. SMRT method showed that the genotype of sample D141966 was −α^3.7^/αα, while by conventional Gap-PCR it was −α^3.7^/−α^3.7^. Validation by MLPA confirmed D141966 had heterozygous −α^3.7^ deletion. To investigate the basis of this discordance, we analyzed the SNV/indels in the αα allele identified by SMRT method and found there were three SNPs in the 3′-terminal of HBA2, which caused dropout of the αα allele in conventional Gap-PCR method that designed primer in this region.

This study found 16 rare *HBA* gene variants. Among them, *c.34A*>*C*, *c.51G*>*C*, *c.84G*>*T*, and *c.19G*>*T* were located in the *HBA1* gene. *c.34A*>*C* and *c.51G*>*C* showed normal hematology, and abnormal hemoglobin was detected [13, 14]. Carriers with *HBA1:c.84G*>*T*, *HBA1:c.19G*>*T*, and *HBA1:c.55G*>*C* genotypes had a normal blood phenotype. When they were compounded with other deletion types, they could be mild or silent [15–17]. Carriers of these gene variants all showed abnormal hemoglobin, and no HbH phenotype was found in the compound Southeast Asian deletion. The --^SEA^/*HBA2*:c.2delT, --^SEA^/*HBA2*:c.2T>C, and --^SEA^/*HBA2*:c.52G>T are located in the more functional *HBA2* gene, causing α chain synthesis to be affected, showing that the non-deletion HbH disease was more serious than the deletion of HbH disease [18–20]. *HBA2*: c.91G>C has a normal phenotype, the main manifestation is abnormal hemoglobin, and HbA2 is reduced [21]. Qadah et al. [22] reported that the *HBA2*: c.−59C>T variant caused a significant reduction in the transcription level of *HBA2* by 53.7%. Our study reported, for the first time, *HBA2*:c.−59C>T and *HBA2*:c.91G>C compound heterozygous cases. Hemoglobin electrophoresis detected abnormal hemoglobin peaks at 3.784 min and 4.349 min, and the routine blood phenotype was normal, due to the abnormal hemoglobin peak time being very close to HbA2, so it could be easily misdiagnosed as a significant increase in HbA2. There are related reports of *HBA2*:c.256G>C [23], but no related reports of *HBA2*:c.256G>A. The phenotype of this case was normal, the main manifestation was abnormal hemoglobin, and HbA2 was reduced.

Among the rare variants in the *HBB* gene, carriers with c.−100G>A, c.−136C>G, c.315+5G>C, c.380T>G, and c.−81A>C were manifested as silent or mild β-thalassemia. The normal hematological phenotype of some cases is consistent with related reports [24–27]. Carrier with c.91A>G was manifested as mild β thalassemia, which is consistent with related reports [28]. Carriers with c.170G>A, c.431A>G, c.232C>T, c.341T>A, and c.431A>G s had normal hematological phenotypes. The content of HbA2 and HbF was within the reference range, and abnormal hemoglobin was detected [29–32]. In the first report of c.341T>A/c.315+5G>C case, abnormal hemoglobin accounted for 93.5%, and HbA was not detected. Carrier with c.431A>G had the peak time of abnormal hemoglobin and HbA2 overlapped, and the content of each component could not be detected correctly. The blood routine examination of c.−248A>G carrier was normal, mainly manifested as a decrease in HbA2 content [33].The phenotype of c.316-45G>C, c.316-179A>C, and c.315+308delA combined with other types of α-thalassemia may be as silent or mild α-thalassemia.

## Conclusions

In summary, in this region, the incidences of rare gene variants and abnormal hemoglobin cases were high. SMRT technology used in the genetic diagnosis of thalassemia had wide detection spectrum with improved efficiency and accuracy over conventional methods. The application of this technology has greatly enriched the thalassemia gene mutation bank and hemoglobin gene profiles in the region, and provided a reference for better prevention and control of thalassemia. However, the pathogenicity of many rare mutant genes is still unclear, and family analysis is required, which brings great challenges to clinical genetic counseling.

## Data Availability

Individual participant data describing the results reported in this article, after de-identification (text, tables, figures, and appendices), together with the study protocol, will be available, beginning 9 months and ending 36 months following article publication. Data will be available for investigators whose proposed use of the data has been approved by an independent review committee identified for this purpose and for individual participant data meta-analysis.
